# Economic Assessment of Supercritical CO_2_ Extraction of Waxes as Part of a Maize Stover Biorefinery

**DOI:** 10.3390/ijms160817546

**Published:** 2015-07-31

**Authors:** Thomas M. Attard, Con Robert McElroy, Andrew J. Hunt

**Affiliations:** Department of Chemistry, University of York, Heslington, York YO10 5DD, UK; E-Mails: tma507@york.ac.uk (T.M.A.); rob.mcelroy@york.ac.uk (C.R.M.)

**Keywords:** supercritical carbon dioxide (scCO_2_), wax, maize stover, cost of manufacture (*COM*), fixed capital investment (*FCI*), extraction, biorefinery

## Abstract

To date limited work has focused on assessing the economic viability of scCO_2_ extraction to obtain waxes as part of a biorefinery. This work estimates the economic costs for wax extraction from maize stover. The cost of manufacture (*COM*) for maize stover wax extraction was found to be €88.89 per kg of wax, with the fixed capital investment (*FCI*) and utility costs (*C*_UT_) contributing significantly to the *COM*. However, this value is based solely on scCO_2_ extraction of waxes and does not take into account the downstream processing of the biomass following extraction. The cost of extracting wax from maize stover can be reduced by utilizing pelletized leaves and combusting the residual biomass to generate electricity. This would lead to an overall cost of €10.87 per kg of wax (based on 27% combustion efficiency for electricity generation) and €4.56 per kg of wax (based on 43% combustion efficiency for electricity generation). A sensitivity analysis study showed that utility costs (cost of electricity) had the greatest effect on the *COM*.

## 1. Introduction

Several methods exist for the extraction of high-value molecules from natural matrices including conventional organic solvent extraction, hydrodistillation, low-pressure solvent extraction, hydrothermal processing and maceration [1,2,3]. However, there has been significant focus on developing clean, “greener” technologies as a result of public-health requirements and consumer demands [1].

Natural waxes are used in a wide variety of industrial applications; with their demand increasing due to the increasing costs of petroleum waxes. A potential feedstock for natural waxes is maize stover. In the United States alone, there is a significant amount of maize stover (as a by-product of maize production) with approximately 68,000,000 tonnes produced every year [4]. Studies have shown that under no-till conditions, two-thirds of the stover can be harvested on a sustainable basis without affecting the soil adversely [5,6,7].

Conventional solvents traditionally utilized in wax extraction (such as hexane) are frequently viewed as being problematic due to their toxicological and environmental hazards [8]. Supercritical fluids typically have properties between those of a liquid and a gas, with the viscosity of a supercritical fluid being an order of magnitude lower than a liquid, while the diffusivity is an order of magnitude higher. This leads to enhanced heat and mass transfer. The solvent can be fine-tuned by varying the temperature and pressure which changes the density of CO_2_ resulting in a change in the density-dependent parameters such as dielectric constant, solubility parameter and partition coefficient. Furthermore supercritical carbon dioxide (scCO_2_) has an easily accessible critical point, is non-flammable, has minimal toxicity and is widely available [9]. ScCO_2_ extraction has been conducted on a commercial scale for over two decades for the extraction of high-value resources from solid substrates [10]. Large-scale as well as small-scale commercial processes have been developed, with the former related to the food industry while the latter is related to more specialized applications. Industrial-large scale extraction processes, in which supercritical carbon dioxide is used, include hop extraction and decaffeination of coffee and black tea leaves [10,11]. Small-scale commercial processes include the removal of pesticides from plant matrices, the extraction of valuable flavoring molecules, essential oils and oleoresins from herbs and spices [12,13]. ScCO_2_ has also been shown to be useful for the extraction of edible-oil however this is currently not a large-scale process due to the fact that the end-product is not of high-value and the process is therefore considered to be non-economically viable [10,14].

ScCO_2_ extraction has been demonstrated to be an ideal clean technology for use as part of a holistic biorefinery [15]. ScCO_2_ has been demonstrated as an effective greener alternative for wax extraction due to its high solvation power, ability to produce extracts that are free of organic residues and high mass transfer rates at relatively low temperatures [8,16,17,18].

In the past, high manufacturing costs associated with supercritical fluid extraction (SFE), resulting from high initial investment costs (associated with high pressure operation/equipment costs) used to be a major stumbling block preventing its use in industrial processes [1,19,20,21]. More recently, there has been significant development of industrial scale units, leading to lower equipment costs associated with SFE processes [21,22]. However, the relatively high pressures needed to reach the supercritical point could make the process energy-intensive and economically non-viable which often restricts the use of scCO_2_ extraction to specialized, high-value applications [23].

Nevertheless, if scCO_2_ extraction is utilized as part of a biorefinery rather than as a stand-alone technology, then this could open doors to further applications (such as extraction of edible oils) as scCO_2_ extraction, besides extracting added value components, has been shown to have a positive effect on the downstream processing of biomass [16]. It is therefore necessary to look at the extraction of compounds by SFE from an economical perspective. Turton *et al.* proposed a methodology to economically assess the cost of producing a desired chemical or chemicals on an industrial scale (Table S1 in supplementary materials) [24]. This methodology has been employed in studies associated with SFE economics and has found to be an effective and appropriate method for evaluating costs of SFE processes, in particular essential oils [1,19,21,25].

To the author’s knowledge, no previous work has been carried out on estimating the economic costs for the extraction of natural waxes using scCO_2_. This work aims to economically assess the supercritical extraction of waxes from maize stover, a biomass residue found in high abundances, using the methodology proposed by Turton *et al.* (for more details about model see supplementary materials). A number of assumptions need to be considered when using this methodology which will be highlighted when appropriate. It should be stated that the supercritical extraction of waxes will be an initial pre-treatment step as part of a biorefinery plant (whereby the maize stover is passed on prior to SFE for downstream processing) and therefore some costs will not be solely attributed to the SFE extraction but to the biorefinery as a whole [16].

## 2. Results and Discussion

### 2.1. Maize Stover Wax Composition

To understand the true value of the wax, it is imperative to know the composition. Therefore, wax characterization was undertaken. In this study, scCO_2_ extraction of waxes from maize stover was conducted on a semi-pilot scale. The % yield of wax extracted was approximately 0.84% which is consistent with previous studies [16]. A plethora of added-value lipophilic molecules were extracted ranging from long-chain fatty acids, *n-*policosanols, fatty aldehydes, *n-*alkanes and wax esters to sterols and steroid ketones. Table 1 summarizes the type and quantity of lipophilic molecules constituting the maize stover wax in this study.

**Table 1:** Quantities of different families of compounds in the scCO_2_ maize stover wax in μg/g of plant.

Compound	Quantity (μg/g of Plant)
Hexanoic acid	1 ± 0.06
Heptanoic acid	0.3 ± 0.07
Octanoic acid	4.1 ± 0.3
Nonanoic acid	3 ± 0.3
Decanoic acid	4.1 ± 0.1
Dodecanoic acid	13.5 ± 0.6
Tetradecanoic acid	23.4 ± 1.1
Pentadecanoic acid	5.2 ± 0.2
Hexadecanoic acid	579 ± 20.9
Heptadecanoic acid	13.5 ± 0.6
Octadecanoic acid	206.2 ± 10.8
Nonadecanoic acid	5.1 ± 0.8
Eicosanoic acid	90.7 ± 5.9
Heneicosanoic acid	11.4 ± 1.6
Docosanoic acid	55.6 ± 4.2
Tricosanoic acid	46.7 ± 3.9
Tetracosanoic acid	76.8 ± 8.3
Pentacosanoic acid	18.1 ± 1.4
Hexacosanoic acid	38.8 ± 4
Octacosanoic acid	6.3 ± 0.8
Total saturated fatty acids	1202.8 ± 65.9
9-hexadecenoic acid	56.5 ± 1.8
C_18_ unsaturated fatty acids	1410.2 ± 82
Total unsaturated fatty acids	1466.7 ± 83.8
Hexacosanol	13.4 ± 1.7
Octacosanol	25.2 ± 3.3
Triacontanol	123.5 ± 9.4
Dotriacontanol	84.7 ± 8.3
Total fatty alcohols	246.8 ± 22.7
Hexacosanal	63.3 ± 6.4
Octacosanal	47.6 ± 2.8
Triacontanal	72.8 ± 8.2
Total fatty aldehydes	183.7 ± 17.4
Pentacosane	2.2 ± 0.1
Heptacosane	9.3 ± 0.4
Nonacosane	24.7 ± 0.9
Hentriacosane	49.2 ± 4.2
Triatriacontane	48 ± 1.6
Total alkanes	133.4 ± 7.2
Campesterol	226.4 ± 9.1
Stigmasterol	319.6 ± 13.6
Β-sitosterol	735.6 ± 15.8
Stigmastanol	226.4 ±9.1
Total Sterols	1358.6 ± 44.3
Stigma-4-*en*-3-one	95.8 ± 2.5
5α-stigmastan-3,6-dione	42.6 ± 3.2
Total steroid ketones	138.4 ± 5.7
Wax ester 40	13.9 ± 1
Wax ester 42	24.9 ± 1.5
Wax ester 43	1.4 ± 0.3
Wax ester 44	29.1 ± 6.5
Wax ester 45	2 ± 0.7
Wax ester 46	23.4 ± 7.8
Wax ester 47	1.4 ± 0.7
Wax ester 48	13 ± 4.2
Wax ester 49	1.5 ± 0.4
Wax ester 50	10.2 ± 2.1
Wax ester 52	5.9 ± 0.5
Wax ester 53	0.8 ± 0.1
Wax ester 54	5 ± 0.5
Wax ester 55	0.5 ± 0.05
Wax ester 56	2.9 ± 0.4
Wax ester 58	1 ± 0.06
Total Wax esters	137.7 ± 26.9
Phytol	8.4 ± 1.1
2-Pentadecanone-6,10,14-trimethyl	90.1 ± 3.7
Total “other” compounds	98.5 ± 4.8

These molecules can be used in a host of applications ranging from nutraceuticals and pharmaceuticals to cosmetics lubricants, polishes and detergent formulations [16,26,27,28,29,30,31,32,33,34,35]. Previous studies have shown the possibility of incorporating the fractionated maize stover wax as a natural defoaming agent in washing machine detergent formulations, replacing non-renewable and environmentally hazardous anti-foaming compounds [16]. Large abundances of unsaturated fatty acids as well as phytosterols were detected in the wax (1466.7 ± 83.8 and 1358.6 ± 44.3 μg/g of plant respectively) which have significant nutraceutical and pharmaceutical properties, including anti-cancer and anti-inflammatory properties as well as lowering LDL-cholesterol levels [26,36,37]. Unsaturated fatty acids are also very useful platform molecules generating a wide variety of other chemicals [38]. The high abundance of these molecules is consistent with previous studies on maize stover wax extraction [16].

Furthermore, in addition to the extraction of high-value waxes, previous studies have shown that scCO_2_ extraction also has a positive effect on the downstream processing of maize stover, enhancing yields for ethanol production [16]. The cellulose, hemicellulose and lignin content of the stover can be found in supplementary materials (Table S2). This highlights the great potential of incorporating scCO_2_ extraction as a first-step in a biorefinery, however, this will be meaningless if the extraction process is not economically viable. Therefore, the cost of manufacture (*COM*) of waxes from maize stover using scCO_2_ was investigated.

### 2.2. Extraction Kinetics

**Figure 1:**
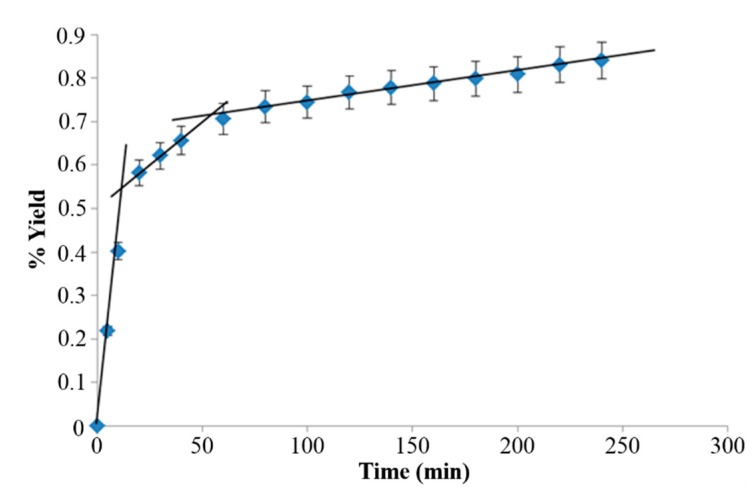
Extraction curve for scCO_2_ extraction of maize stover.

The extraction time was determined by investigating the SFE extraction kinetics using a laboratory-scale supercritical unit. The extraction was carried out for 4 h, collecting samples at specific time intervals. It is assumed that the performance of the industrial scale unit should be the same or very similar to that of the laboratory supercritical unit. This should not be a problem if the bed density, particle size and the ratio between the mass of the solid and the CO_2_ flow rate are kept constant. Figure 1 illustrates the % yield obtained in this study for the extraction of wax from maize stover as a function of time.

Typically, in an SFE process there are three linear regions in the extraction curve profiles; the constant extraction rate (CER) which corresponds to the extraction of solute molecules that are easily accessible and therefore convection in the solvent film surrounding the biomass particles dominates the mass transport, the falling rate period (FER) where both convection and diffusion effects play a role in mass transport and the third line which corresponds to a process that is entirely diffusion-controlled (in this part of the extraction curve, the extraction rate is very low) [39]. The maximum extraction rates are normally observed at the CER region and it is therefore necessary, from an economical perspective, to identify the CER region for extraction of solutes from maize stover. The total yield extracted after 4 h was found to be 0.84%. After 40 min of extraction (the end of the FER region), 78% of the total wax is extracted.

### 2.3. Economic Assessment of Maize Stover Wax Extraction: Cost of Manufacture (COM)

The *COM* of extractives involves three main types of costs; direct Costs (*DC*) (operational costs which are dependent on the production (manufacturing) rate and include raw material costs, operational labour, utilities among others), fixed costs (*FC*) (not dependent on production rate and include territorial taxes, insurance, depreciation *etc.*) and general expenses (*GE*) (cover business maintenance and consist of management, administrative sales, research and development costs *etc.*). These three components of the *COM* are estimated in terms of five main costs: fixed capital investment (*FCI*), cost of operational labour (*C*_OL_), cost of utilities (*C*_UT_), cost of waste treatment (*C*_WT_) and cost of raw materials (*C*_RM_).

The *COM* of wax extraction with depreciation was calculated using the following equation [24]:

(1) $$COM=0.280\times FCI+2.73\times C_{\text{OL}}+1.23\times (C_{\text{RM}}+C_{\text{WT}}+C_{\text{UT}})$$

#### 2.3.1. Fixed Capital Investment *FCI*

A typical industrial supercritical extraction unit (used in the extraction of spices, natural pigments, nutraceuticals *etc.*) is composed of two 0.4 m^3^ extractors, a series of flash tanks (for fractionation), a CO_2_ reservoir, a CO_2_ pump (for compression of the solvent) and a CO_2_ heater. The cost of the industrial scale unit is around € 1,400,000 [21,40]. On a yearly basis, the fraction of investment is calculated by multiplying the total investment by the depreciation rate. The depreciation rate is assumed to be 10% per year and is used in the calculation of the *COM*. Another part of the investment is the initial quantity of CO_2_ that is required to fill the CO_2_ reservoir; however this cost is generally negligible when comparing it to the extraction unit cost.

#### 2.3.2. Operational Labour Costs (*C*_OL_)

In terms of man-hour per operation-hour, the total *C*_OL_ is estimated by using tables which are presented by Ulrich (1984). The total time when the extraction columns are under operation was taken to be 330 days per year of continuous 24 h per day shift which corresponds to 7920 h of continuous extraction. It is assumed that, in the industrial SFE unit there will be two operators per shift and the *C*_OL_ was taken to be € 3.00/h. This value is solely attributed to the work that the operators will carry out on the SFE of waxes. The operators will have other duties within the biorefinery and their overall wage would therefore be higher.

#### 2.3.3. Raw Material Costs (*C*_RM_)

Raw material costs for SFE include the solid substrate containing the solute to be extracted as well as the CO_2_ that is lost in the extraction process. The cost of the former includes the price of the biomass itself as well as all the cost of all the pre-processing steps leading to the final biomass product used in the extraction such as drying, comminution and cleaning. Since wax extraction from maize comes from the waste following harvesting of the grain, *i.e.*, from the corn stover (stalk, leaves, cob and husk tissues), the *C*_RM_ in this study focuses on the costs of harvesting and supplying corn stover to biorefineries. Since maize stover has significant promise for the production of bioenergy, studies have been carried out on costs of corn stover. It is challenging to estimate an appropriate *C*_RM_ for stover as an extensive literature search showed a large variation in the stover *C*_RM_ (Table 2)_._

**Table 2:** Estimates of maize stover *C*_RM_ found in literature [4,5,41,42,43,44,45,46,47,48,49,50,51,52,53,54].

Study	Cost of Stover
Perlack *et al.*, 2003 [41]	$43.10–$56.10/dry metric tonne (Mid-point $49.60)
Eggeman *et al.*, 2005 [42]	$35/dry metric tonne
Graham *et al.*, 2007 [5]	$33/dry metric tonne
Sendich *et al.*, 2008 [43]	$40/dry metric tonne
Dutta *et al.*, 2009 [44]	$60.10/dry metric tonne
Sokhansanj *et al.*, 2010 [45]	$74/dry metric tonne (baled), $84/dry metric tonne (chopped) and $86/dry metric tonne (pelletised) (assumed pelletised in this calculation)
Kazi *et al.*, 2010 [46]	$83/dry tonne
Humbird *et al.*, 2011 [47]	$58.50/dry tonne
Gonzalez *et al.*, 2012 [48]	$80.3/dry tonne
Fiegel *et al.*, 2012 [49]	$85.40/dry tonne
Vadas *et al.*, 2013 [51]	$44.09/dry tonne (most expensive)
Tao *et al.*, 2013 [52]	$58.50/dry tonne
Meyer *et al.*, 2013 [50]	$58.50/dry tonne
Petrou *et al.*, 2014 [53]	$58.50/dry tonne
Ou *et al.*, 2014 [54]	$83/dry tonne
Thompson *et al.*, 2014 [4]	$88.19/dry tonne

In order to determine the effect of the price of the biomass three different calculations based on three different *C*_RM_ values were carried out: the average *C*_RM_ obtained from all studies ($62.61/dry tonne equivalent to €55.92 per·odt^−1^, the highest *C*_RM_ ($88.19/dry tonne equivalent to €78.76 per·odt^−1^) and the lowest *C*_RM_ ($33/dry metric tonne equivalent €29.47 per·odt^−1^).

#### 2.3.4. Cost of Waste (*C*_WT_)

In an industrial SFE unit, the CO_2_ is recycled and therefore the only waste involved in the process is the CO_2_ which leaks from the system and the exhausted solid. The former is negligible while the exhausted stover biomass can be utilized further downstream as part of a biorefinery process (or incorporated back into the soil for the uptake of nutrients). Therefore it can be assumed that little or no waste is generated during the extraction process. Therefore the *C*_WT_ can be ignored.

#### 2.3.5. Cost of Utilities (*C*_UT_)

Three types of costs are involved in the *C*_UT_; the costs associated with the electric power used in the CO_2_ pump, the costs associated with the CO_2_ heater and costs associated with refrigeration.

##### Costs Associated with the Electric Power Used in the CO_2_ Pump

In order to calculate electric power costs for the CO_2_ pump the pressure and temperature applied during the extraction process as well as the extraction time were determined.

Previous studies investigated the optimal conditions for maize wax extraction using scCO_2_, where the highest wax yields were obtained with a pressure of 400 bar and 65 °C [16]. The pressure and temperature utilized in the extraction process give the specific enthalpy, from which the total energy used in the extraction process can be obtained by multiplying the variation of specific enthalpy by the extraction time and the CO_2_ mass flow rate. In the case of maize stover, the specific enthalpy of CO_2_ using a pressure of 400 bar and 65 °C is 314.11 kJ/kg [55].

When analyzing the extraction kinetics together with the cost of raw materials and total wax which could be extracted per day, it was found that it is more profitable to carry out 40 min extractions (gives a higher overall wax yield per day and reduces the overall costs significantly) when compared to 1 h extractions and therefore 40 min was selected as the time for each extraction.

The experimental bed density of the maize stover was found to be 0.33 g/cm^3^, corresponding to 132 kg of maize stover biomass per extraction on an industrial scale unit. The CO_2_ mass flow rate required for the industrial-scale unit would be approximately 2964.1 kg/h (based on the CO_2_ flow rate in the laboratory-scale extraction which was 6.7 × 10^−4^ kg/s). The cost of electricity was assumed to be €0.112/kwh [56].

(2) Pure CO2 enthalpy at 400 bar, 65°C=314.11 kJ/kg CO21 kg of CO2=314.11 kJ CO22964.1 kg of CO2=931,053.45 kJ CO2P(kw)=E(kJ)tsP(kw)=931,053.453600=258.6 Kw·h−1Cost of electricity (UK)=€0.112 per Kw·h−1Cost of electricity =258.6 ×0.112= €28.97/h

The costs associated with the CO_2_ pump were calculated to be €28.97/h.

##### Costs Associated with the CO_2_ Heater

The CO_2_ has to be heated from 4 °C (temperature of CO_2_ in the pumps) to 65 °C. The mass of carbon dioxide used per hour is 2875.1 kg, the C_p_ of carbon dioxide at 65 °C is 0.88 kJ·kg^−1^·k^−1^ and the ∆T is 61 °C. Therefore the heat required (in MJ), Q, was calculated as follows:

(3) $$\begin{array}{ccc} Q & = & \frac{{\text{MC}}_{\text{p}}\Delta \text{T}}{\text{efficiency}}\;(\text{Assume }50\text{\% efficiency})\\ Q & = & 2964.1\;\times 0.88\times 61/0.5\\ Q & = & 318,225.8\text{ KJ per hour}\;(318.2\;MJ) \end{array}$$

Thus the heat energy required is 318.2 MJ per hour. A number of studies have looked into the calorific content of corn stover. An average value from these studies was taken and it is assumed that the energy that is given off when burning dry maize stover is 17.4 MJ·kg^−1^ [57,58,59,60,61]. Therefore the amount of maize stover that is required is 18.3 kg per hour which is only around 13.9% of the biomass that is used in each extraction. Therefore the energy which is required to heat the extractor may be obtained by burning 13.9% of the biomass that is loaded into the extractor and the costs that are associated with heating the extractors are thus negligible.

##### Costs Associated with Refrigeration

A typical refrigeration cycle comprises of a working fluid circulated around a loop which is made up of a compressor, evaporator, expansion valve or turbine and condenser. Refrigeration is more expensive than heating since it requires electrical power. The water has to be cooled from 20 °C (around room temperature) to 4 °C. In order to determine the refrigeration costs the energy required for refrigeration must be determined by calculating the coefficient of performance, *COP*.

(4) $$\begin{array}{ccc} Q & = & {\text{MC}}_{\text{p}}\Delta \text{T}\\ COP\;20^{\text{o}}\text{C} & = & 0.08\\ COP\;4^{\text{o}}\text{C} & = & 0.15\\ Q & = & (2964.1\;\times 0.846\times 16)\;\times \;\frac{0.15}{0.08\;}\;=75,228.86\\ P & = & \frac{75,228.86}{3600}=20.90\text{ Kw}\cdot {\text{h}}^{-1}\\ & = & 20.90\text{ Kw}\cdot {\text{h}}^{-1}\times 0.112\text{ €}\cdot \text{Kw}\cdot {\text{h}}^{-1}=\text{ €}2.34/\text{h} \end{array}$$

The costs associated with refrigeration are €2.34 per hour of extraction.

Therefore it is assumed that the total utility costs, *C*_UT_ are €31.31 per hour.

#### 2.3.6. Total *COM* Calculation

Taking all calculations into account the *COM* for the supercritical extraction of waxes from maize stover assuming the average *C*_RM_ of €55.92 per·odt^−1^ are:

(5) $$\mathrm{COM}=0.280\times \mathrm{FCI}+2.73\times C_{\text{OL}}+1.23\times (C_{\text{RM}}+\;C_{\text{WT}}+C_{\text{UT}})\mathrm{COM}=0.280\times \left(1,400,000\right)+2.73\times \left(47,250\right)+1.23\times \left(87,691.51+\;0+247,947.96\right)\mathrm{COM}=\frac{€934,566.2/\text{year}}{1568.16\text{ tonne}/\text{year}}\mathrm{COM}=€596/\text{tonne of maize stover}\mathrm{COM}=€88.89/\text{kg of maize stover wax}$$

The final *COM* was found to be €596 per tonne of maize stover biomass or €88.89 per kg of wax. If the lowest *C*_RM_ was taken into account of €29.47 per·odt^−1^ the final *COM* would come up to €563 per tonne of maize stover biomass or €84.03 per kg of wax, while if the highest *C*_RM_ was used of €78.76 per·odt^−1^ the final *COM* would come up to €624 per tonne of maize stover biomass or €93.08 per kg of wax.

**Figure 2:**
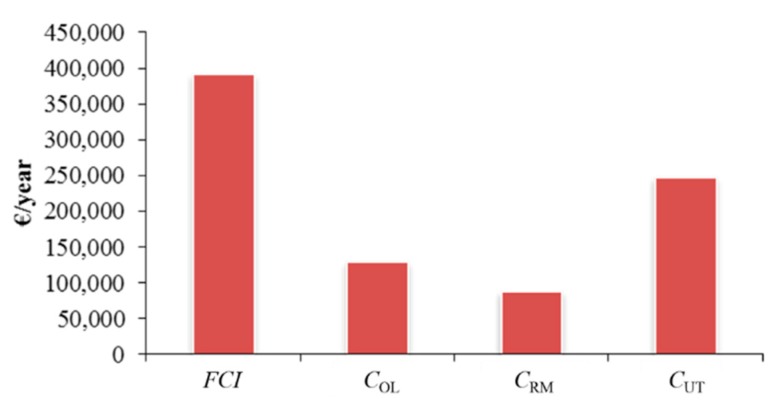
Distribution of costs in the extraction of maize stover wax.

As can be seen from Figure 2, the main contributors to the *COM* are the *FCI* and the *C*_UT_. The main cost for the *C*_UT_ is the electricity that is required to pump the CO_2_ at the required pressure and temperature. Raw material costs and labour costs contribute less to the *COM*. This value is only an estimate and is based on a number of assumptions. The costs can be improved by varying some of the parameters. First of all the figure for the amount of biomass that can be loaded into the supercritical extractor was based on milled biomass. In industry, biomass is normally received as pellets (pelletized) and this increases the biomass loading by three times [62]. If pelletized biomass was used (and assuming an average C_RM_ of €55.92 per·odt^−1^):

(6) $$\mathrm{COM}=0.280\times (1,400,000)+2.73\times (47,250)+1.23\times \left(263,074.52+\;0+743,843.88\right)\mathrm{COM}=\frac{€1,760,239.2/\text{year}}{\;4704.48\text{ tonne}/\text{year}}\mathrm{COM}=€374.2/\text{tonne of maize stover}\mathrm{COM}=€57.1/\text{kg of maize stover wax}$$

Therefore, if pelletized maize stover is taken into account the *COM*/tonne of maize stover has dropped significantly, by €347.2 while the *COM* of wax (per kg) has dropped by €31.79, *i.e.*, significant savings. Furthermore, in this study maize stover was used in the process, yielding 0.8% wax. Maize leaves have a greater wax content than maize stover. If the maize leaves were used, then the wax yield is almost 2.2 times as much (1.74%) [63].

(7) $$\mathrm{COM}=\frac{€1,760,239.2/\text{year}}{81,857.952\text{ kg wax}/\text{year}}\mathrm{COM}=€21.50/\text{kg of maize leaf wax}$$

This is a four-fold reduction in cost compared to stover wax. Finally, it was assumed in the calculations that the cost of raw materials (*C*_RM_) is solely for the supercritical extraction. As stated previously, the supercritical extraction is only the first step (pre-treatment step) in a biorefinery and thus the biomass will be passed on within the biorefinery for further processing. The cost of raw materials must also be shared throughout the entire processes within the biorefinery. The *C*_RM_ value would therefore be lower leading to lower overall manufacturing costs (*COM*).

#### 2.3.7. Utilization of Maize Stover Biomass for Electricity Generation

Since the supercritical extraction of waxes would be carried out as part of a biorefinery set-up, the maize stover biomass collected after the extraction would be passed on to the next stage of the biorefinery process and hence further lower the *COM* of the wax. The least elegant and therefore lowest added value-step, would be to simply burn the waste biomass for energy recovery. Herein, cost estimations for electricity generation were carried out as an example of downstream processing of the biomass.

Different technologies have different energy conversion efficiencies from biomass. However, intense development is occurring within this area and a number of highly efficient technologies are emerging. The greatest efficiency was found to be 43% [64], while the average of all available technologies is 27% [65]. Therefore two calculations were carried out: one based on the technology with the greatest efficiency while the other based on the average efficiency of all technologies.

##### Calculation Assuming Use of Most Efficient Technology (combustion)

(8) $$\begin{array}{l} \text{Mass of milled maize per exraction }\left(40\text{ min}\right):132\text{ kg}\\ \text{Mass of pelletised maize per extraction}:132\times 3=396\text{ kg}\\ \text{Mass of wax extracted }\left(\text{assuming }1.74\text{\% yield}\right):396\times 0.0196=6.89\text{ kg} \end{array}$$

Therefore maize biomass after each extraction:

(9) 396 kg−6.89 kg=389.11 kg of maize

When heating the extractors, 54.87 kg of maize is required for each extraction:

(10) 389.11 kg−54.87 kg=334.24 kg of available maize per extraction

Energy of combustion for maize:

(11) $$\begin{array}{ccc} 1\text{ kg of maize} & = & 17.4\text{ MJ}\\ 17.4\text{ MJ }\times 334.24 & = & 5815.78\text{ MJ}/\text{extraction }(40\text{ min})\\ & = & 8723.66\text{ MJ}/\text{h} \end{array}$$

Assuming 43% efficiency:

(12) P(kw)=E(MJ)tsP(kw)=(8723.66)3.6 ×0.4=1041.99 Kw·h−1Cost of electricity (UK)=€0.112 per Kw·h−1Value of electricity generated per extraction=1026.38 Kw·h−1×0.112 € Kw·h−1=€116.70Value of electricity generated per tonne of maize=€116.70×1000396=€294.71 per tonne of maize =€16.94 per kg of wax

Therefore when subtracted from the cost of wax production:

(13) Total COM cost=21.50−16.94= €4.56 per kg of wax

##### Calculation Assuming Average Efficiency of All Technologies

**Figure 3:**
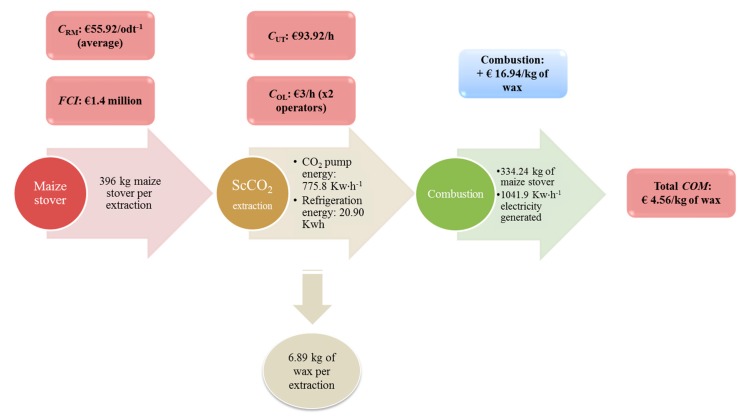
Schematic highlighting the energy and material inputs for maize leaf wax.

When carrying out the same calculation above using the average efficiency of all technologies (27% efficiency), the total *COM* per kg of wax was found to be €10.87 per kg of wax. Therefore if the biomass was to be utilised after the extraction for electricity production, the *COM* of the maize wax would decrease to €10.87 per kg of wax when taking the average energy efficiency of all available technologies (27%), while the cost is €4.56 for every kg of wax when the most efficient technology is taken into consideration. The inclusion of a more high value step within the biorefinery such as microwave pyrolysis of the biomass prior to energy recovery or fermentation of the stover for production of ethanol and surfactants, would further reduce the *COM* [15,16]. A detailed spreadsheet (entitled Economics COM calculations) containing all of the calculations may be found in supplementary materials (Table S3). Figure 3 is a schematic that summarizes the key results and highlights all the relevant material and energy inputs.

#### 2.3.8. Sensitivity Analysis

**Figure 4:**
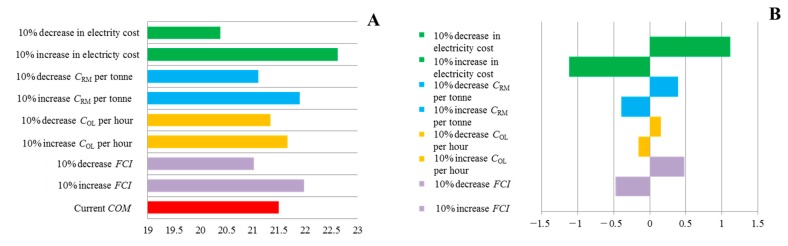
Cost of manufacture (*COM*) kg^−1^ of maize wax leaf pellets: (**A**) Difference in *COM* (€) when varying the different parameters (electricity cost, *C*_RM_, *C*_OL_ and *FCI*) by 10% (increase or decrease); and (**B**) % difference in *COM* when varying the different parameters by 10% (increase or decrease).

In order to highlight the most relevant parameters, a simple one-at-a-time sensitivity analysis was carried out, whereby different parameter values were varied in order to identify the most relevant parameters. A 10% increase or decrease in the cost of each parameter (*C*_RM_, *C*_UT_, *C*_OL_ and *FCI*) was implemented to observe the effect on the overall *COM*. The results from the study (Figure 4A,B) indicate that the major parameter having the greatest effect on the overall *COM*, was found to be the *C*_UT_ costs, *i.e.*, the electricity costs associated with the CO_2_ pump and refrigeration while varying the *C*_OL_ by 10% had little effect on the overall *COM*.

## 3. Experimental Section

### 3.1. Material

The maize stover was harvested after R6 stage (silage) from plants cultivated under field conditions near York (UK). The cobs were removed and the stover samples were milled to 0.5 cm particles with a hammer mill. They were then dried in a convection oven at 60 °C for 24 h. The biomass was removed, weighed and placed in the convection oven once more. At specific intervals the biomass was weighed until a constant weight was achieved.

### 3.2. Supercritical Fluid Extraction of Maize Stover Wax for Analysis

The supercritical carbon dioxide extractions were carried out using a SFE-500 provided by Thar technologies (Pittsburgh, PA, USA). Supercritical fluid grade carbon dioxide (99.99%) was used to conduct the extractions. 100 g of milled biomass (maize stover) was placed into the 500 cm^3^ extraction vessel and connected to the extraction system. The required temperature and pressure were applied. The reaction vessel was heated to 50 °C and 5 min were allowed for it to equilibrate. An internal pump was used in order to obtain the required pressure (400 bar). The system was run in dynamic mode, in which the carbon dioxide which contained the epicuticular lipids, was allowed to flow into the collection vessel. A flow rate of 40 g·min^−1^ of liquid CO_2_ was applied and the extraction was carried out for 4 h. When the extraction was terminated, depressurisation of the system was carried out over a period of 4 h. The wax was collected by rinsing the collection vessel twice with approximately 100 cm^3^ of DCM. The solvent was removed *in vacuo*. The crude wax product was weighed and the % yield was calculated. The plant material was removed and a brush was used to clean the extraction vessel. The system was washed in dynamic mode using a combination of supercritical carbon dioxide and ethanol (10%) for 45 min at the extraction pressure. The pump supplying the modifier was then turned off and carbon dioxide was allowed to pass through the system for an additional 20 min.

### 3.3. Supercritical Extraction of Maize Stover Extraction Kinetics

100 g of milled biomass (maize stover) was placed into the 500 cm^3^ extraction vessel and connected to the extraction system (Thar technologies, Pittsburgh, PA, USA). The required temperature and pressure were applied. The reaction vessel was heated to 65 °C and 5 min were allowed for it to equilibrate. An internal pump was used in order to obtain the required pressure (400 bar). The system was run in dynamic mode, in which the carbon dioxide which contained the epicuticular lipids was allowed to flow into the collection vessel. A flow rate of 40 g·min^−1^ of liquid CO_2_ was applied. Samples of the extract were collected every 5 min for the initial 20 min and the mass of each sample was recorded. Samples of the extract were then collected every 20 min for the next 3 h and 40 min, so that a total extraction time of 4 h was implemented. The mass of each sample was recorded.

### 3.4. Derivitisation Prior to HT-GC (High Temperature-Gas Chromatography) Analysis

Thirty mg of crude wax extract were silylated by adding 200 μL *N*,*O*-*bis*-(trimethylsilyl)-trifluoro-acetamide and 100 μL toluene. The closed vial was heated in an oven for 30 min at 75 °C.

### 3.5. HT-GC Procedure for Analysis of Wax

HT-GC analysis was performed on an Agilent Technologies 6890N Network GC System. A ZB-5HT capillary column (30m × 250 μm × 0.25 μm nominal) was fitted at constant pressure of 22.35 psi. The carrier gas used was helium. The injector temperature and the flame ionisation detector temperature were maintained at 300 °C. The samples were injected by automated injection (1 μL injection volume) with a split ratio of 5:1. An initial oven temperature of 60 °C was maintained for 1 min. The temperature was increased at a ramp rate of 8 °C·min^−1^ until 360 °C.

Quantification of the lipid components was carried out by means of internal standard calibration and response factors (*R*_f_). Seven point linear calibration graphs were produced using external standards for the quantification of hydrophobic compounds.

### 3.6. HT-GC-MS (High Temperature-Gas Chromatography Mass Spectrometry) Procedure for Analysis of Wax

HT-GC-MS was performed on a Perkin Elmer Clarus 500 GC coupled with a Clarus 500 quadrupole mass spectrometer. This was fitted with a DB5HT capillary column (30 m × 250 μm × 0.25 μm nominal) at constant pressure of 22.35 psi. The carrier gas used was helium. The temperature of the injector was 360 °C and the flow rate was set to 1.00 mL/min. The initial oven temperature was maintained at 60 °C for 1 min. The temperature was then ramped at a rate of 8 °C·min^−1^ until 360 °C and held for 30 min. The Clarus 500 quadrupole mass spectra was operated in the electron ionisation mode (EI) at 70 eV, a source temperature of 300 °C, quadrupole at in the scan range of 30–1200 amu per second.

Another method was developed for the analysis of wax esters. The temperature of the injector was 380 °C and the flow rate was set to 1 mL/min. The initial oven temperature was maintained at 100 °C for 1 min. The temperature was then ramped at a rate of 10 °C·min^−1^ until 380 °C and held for 20 min. The Clarus 500 quadrupole mass spectra (Perkin Elmer, Waltham, MA, USA) was operated in the electron ionisation mode (EI) at 70 eV, a source temperature of 300 °C, quadrupole at in the scan range of 30–1200 amu per second. The data was collected with the PerkinElmer enhanced TurboMass (Ver5.4.2) chemical software (Perkin Elmer, Waltham, MA, USA) and compounds were identified by comparison of mass fragmentation patterns with spectra contained in the National Institute of Standards and Technology (NIST) library (version 2.2) and by direct comparison with standard compounds.

## 4. Conclusions

This is the first time a techno-economic assessment for the supercritical extraction of waxes from biomass has been carried out. The cost of manufacture (*COM*) for maize stover was found to be €88.89/kg of wax. The cost of extracting wax from maize can be lowered if: (i) the biomass is pelletised; (ii) the extraction is carried out on the leaves; and (iii) if the biomass is combusted post extraction. This gives a *COM* of €10.87 per kg of wax (based on 27% combustion efficiency for electricity generation) and €4.56 per kg of wax (based on 43% efficiency). It must be stated that these costs are estimated for an industrial supercritical plant with a yearly capacity of around 1600 tonne of biomass. A more elegant biorefinery scenario incorporating hydrolysis and fermentation or microwave pyrolysis of the biomass could lead to the generation of other added-value products prior to combustion of residual material [15,16]. This study has shown that, if certain parameters are taken into account, a high value product (wax) can be obtained for a low price, when thinking holistically.

## Supplementary Materials

Supplementary materials can be found at http://www.mdpi.com/1422-0067/16/08/17546/s1.
