# Supplementary material for: Economic Assessment of Supercritical CO2 Extraction of Waxes as Part of a Maize Stover Biorefinery
Source: Int J Mol Sci. 2015 Jul 31;16(8):17546–64. doi: 10.3390/ijms160817546 (PMC4581208; doi:10.3390/ijms160817546)
Supplement: Supplementary file 1 [file ijms-16-17546-s001.zip › ijms-89052-Supplementary Information/ijms-89052-Supplementary Information.pdf]

Supplementary Information

S1. Cost of Manufacturing (*COM*).

When calculating manufacturing costs of a chemical product (such as extractives), three main types of expenses are involved:

(i) Direct Costs (*DC*): Direct costs deal with the operational costs. They are dependent on the production (manufacturing) rate and include raw material costs, operational labour, utilities among others.

(ii) Fixed costs (*FC*): These are not dependent on production rate and include territorial taxes, insurance, depreciation and so on. They are charged at constant rates even when the plant is not operational.

(iii) General expenses (*GE*): The general expenses cover business maintenance and consist of management, administrative sales, research and development costs *etc*.

$$COM = DC + FC + GE \quad (1)$$

Therefore, the cost of manufacturing (*COM*) is the sum of direct costs, fixed costs and general expenses. These three components of the *COM* are estimated in terms of five main costs:

(1). Fixed capital investment (*FCI*);
(2). Cost of operational labour (C_{OL});
(3). Cost of utilities (C_{UT});
(4). Cost of waste treatment (C_{WT});
(5). Cost of raw materials (C_{RM}).

Table S1. Data given to estimate individual cost items.

Cost Item	Typical Range of Multiplying Factors	Value Used in Text
	(1) **Direct Costs**	
A. Raw Materials	C_{RM}	Must be calculated
B. Waste treatment	C_{WT}	Must be calculated
C. Utilities	C_{UT}	Must be calculated
D. Operating labour	C_{OL}	Must be calculated
E. Direct supervisory and clerical labour	(0.1–0.25)C_{OL}	0.18C_{OL}
F. Maintenance and repairs	(0.02–0.1)*FCI*	0.06*FCI*
G. Operating supplies	(0.1–0.2)Line1.F	0.009*FCI*
H. Laboratory charges	(0.1–0.2)C_{OL}	0.15C_{OL}
I. Patents and royalties	(0–0.06)C_{OM}	0.03C_{OM}
Total Direct Costs	C_{RM} + C_{WT} + C_{UT} + 1.33C_{OL} + 0.03*COM* + 0.069*FCI*	
	(2) **Fixed Costs**	
A. Depreciation	0.1*FCI*	0.1*FCI*
B. Local taxes and insurance	(0.014–0.05)*FCI*	0.032*FCI*
C. Plant overhead costs	(0.5–0.7)(Line 1.D. + Line 1.E + Line 1.F)	0.708C_{OL} + 0.036*FCI*
Total Fixed Costs	0.708C_{OL} + 0.068*FCI* + depreciation	
	(3) **General Expenses**	
A. Administration costs	(0.15)(Line 1.D. + Line 1.E + Line 1.F)	0.177C_{OL} + 0.009*FCI*
B. Distribution and selling costs	(0.02–0.2)*COM*	0.11*COM*
C. Research and development	0.05*COM*	0.05*COM*
Total General Expenses	0.177C_{OL} + 0.009*FCI* + 0.16*COM*	
Total Costs (*COM*): C_{RM} + C_{WT} + C_{UT} + 2.215C_{OL} + 0.190*COM* + 0.146*FCI* + depreciation		

Line 1.D. = C_{OL}; Line 1.E = (0.1–0.25)C_{OL}; Line 1.F = (0.02–0.1)*FCI*.

Other individual items can be calculated using equations that are displayed in Table S2. There is a typical range for constants (multiplication factors) for each equation, which are needed to estimate each of these individual costs. The mid-point value for each range is used for assessing the costs where no available information is given.

When utilizing the midpoint values shown in Table S2, three equations can be generated for each category:

$$\begin{aligned} DC &= C_{\mathrm{RM}} + C_{\mathrm{WT}} + C_{\mathrm{UT}} + 1.33C_{\mathrm{OL}} + 0.069FCI + 0.03COM \\ FC &= 0.708C_{\mathrm{OL}} + 0.068FCI + depreciation \\ GE &= 0.177C_{\mathrm{OL}} + 0.009FCI + 0.16COM \end{aligned} \quad (2)$$

Addition of these categories and solving for *COM* gives the total *COM* such that:

$$COM = 0.180FCI + 2.73C_{OL} + 1.23(C_{\mathrm{RM}} + C_{\mathrm{WT}} + C_{\mathrm{UT}}) \quad (3)$$

The above equation is the COM without depreciation. In order to calculate the *COM* with depreciation, 0.1 *FCI* has to be added. Therefore, the final equation is:

$$COM = 0.280FCI + 2.73C_{\mathrm{OL}} + 1.23(C_{\mathrm{RM}} + C_{\mathrm{WT}} + C_{\mathrm{UT}}) \quad (4)$$

S2. Compositional Analysis of Stover.

Table S2. Compositional Analysis of Stover.

Compounds	% Composition
Crystalline cellulose	21.56
Hemicellulose	15.23
Lignin	25.62
Ash	4.83
